# NCOR1 may be a potential biomarker of a novel molecular subtype of prostate cancer

**DOI:** 10.1002/2211-5463.13004

**Published:** 2020-11-08

**Authors:** Lu Tang, Lixia Zhang, Lei Liu, Liping Dong, Yuan Dong, Wenhe Zhu, Huiyan Wang

**Affiliations:** ^1^ Jilin Collaborative Innovation Center for Antibody Engineering Jilin Medical University Jilin China; ^2^ School of Landscape Jiangxi Agricultural University Nanchang China

**Keywords:** molecular subtype, NCOR1, prostate cancer

## Abstract

Prostate cancer (PCa) is the most frequently diagnosed male cancer. An earlier study of a cohort of 333 primary prostate carcinomas showed that 74% of these tumors fell into one of seven subtypes of a molecular taxonomy defined by specific gene fusions (*ERG*, *ETV1/4* and *FLI1*) or mutations (*SPOP*, *FOXA1* and *IDH1*). Molecular subtypes may aid in distinguishing indolent cases from aggressive cases and improving management of the disease. However, molecular features of PCa outside the seven subtypes are still not well studied. Here we report molecular features of PCa cases without typical features of the established subtypes. We performed comprehensive genomic analysis of 91 patients, including 54 primary and 37 metastatic cases, by whole‐exome sequencing. *TP53*, *SPOP*, *FOXA1*, *AR* (androgen receptor) and a *TMPRSS2*–*ERG* fusion emerged as the most commonly altered genes in primary cases, whereas *AR*, *FOXA1*, *PTEN*, *CDK12*, *APC* and *TP53* were the most commonly altered genes in metastatic cases. Nuclear receptor corepressor (*NCOR1*) genomic alterations have been identified in 5% of cases, which are nontypical molecular features of PCa subtypes. A novel *NCOR1* c.2182G>C (p.Val728Leu) was identified in tumor. RT‐PCR was used to show that this mutation caused loss of *NCOR1* exon 19 and might be oncogenic in PCa. NCOR1 is involved in maintenance of mitochondrial membrane potential in PCa cells, and loss of NCOR1 might contribute to PCa progression. Therefore, NCOR1 may be a potential molecular marker of a subtype of PCa.

## Graphical abstract

Our data suggest that Nuclear receptor corepressor (*NCOR1*) is involved in the maintenance of mitochondrial membrane potential in prostate cancer cells, and loss of *NCOR1* may contribute to prostate cancer progression.

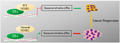

**Abbreviations**
ΔΨm: measurement of mitochondrial membrane potential
CRPC: recurrent castration‐resistant prostate cancer
DCFH‐DA: 2′,7′‐dichlorofluorescin diacetate
FFPE: formalin‐fixed paraffin‐embedded
EMT: epithelial mesenchymal transition
H&E: hematoxylin and eosin staining
HSF: human splicing finder
NC: negative control
NCOR1: Nuclear receptor corepressor
PCa: prostate cancer
ROS: reactive oxygen species
SNV: single‐nucleotide variant
SSF: splicing site finder
TCGA: The Cancer Genome Atlas
VUS: variant with unknown significance

Prostate cancer (PCa) is the most frequently diagnosed male cancer, with an estimated 164 690 cases in 2018 in the United States [1]. Many factors, including genetic and demographic factors, such as genetic variation and susceptibility, family history, age and race, contribute to the high incidence of PCa [2]. Localized PCa is the most common status at the time of the cancer diagnosis and is highly variable, either indolent to be safely observed or aggressive to metastasis and final death from the disease. It is still a major obstacle to distinguish indolent from aggressive PCa and predict outcome, despite the application of established multiple‐risk stratification systems. Next‐generation sequencing is being used in large‐scale whole‐exome sequencing and whole‐genome sequencing to identify clinically actionable mutations. The identified molecular features might potentially be applied to establish further risk stratification to help distinguish indolent from aggressive PCa, as well as commitment to precision medicine.

Molecular subtypes of PCa are further risk stratifications based on molecular and genetic profiles. A study of a cohort of 333 primary prostate carcinomas showed that 74% of these tumors fell into one of seven subtypes of a molecular taxonomy defined by specific gene fusions (E*RG* *,* *ETV1/4* and *FLI1*) or mutations (*SPOP* *,* *FOXA1* and *IDH1*) [3]. Fusion of the *ETS* family of genes is the most common recurrent rearrangement in PCa. *TMPRSS2–ERG* fusion accounted for 40–50% of the patients diagnosed with PCa, while *TMPRSS2* fused to *ETV4*, *ETV5* and *ETV1* has also contributed to 1–5% of PCas [4, 5]. Three subtypes were classified by mutations in *FOXA1*, *SPOP* and *IDH1* [3, 6, 7]. Upon progression of localized PCa to metastatic disease, *PTEN* and *TP53* are the two genes that contribute the most driver mutations for PCa to be aggressive and metastatic [8]. *RB1* loss (28%), amplification of *MYC* (10%), mutations in *ATM* (19%) and *BRACA2* (~ 7%) are less common genetic alterations in the progression of PCa [9].

Molecular subtypes may result in distinguishing indolent cases from aggressive cases and improving management of the disease. However, molecular features of PCa outside the seven subtypes are still not well studied. Here we reported molecular features of PCa cases without typical features of the established subtypes.

## Materials and methods

### Patient samples and sequencing analysis

Patient samples were derived from patients with high‐grade (Gleason score 6 or higher) or metastatic biopsies. All subjects included in this study provided informed written consent for research use of tumor tissue with institutional review board approvals or appropriate waivers. Formalin‐fixed paraffin‐embedded (FFPE) tissue specimens or flash‐frozen needle biopsies and matched normal samples underwent nucleic acid extraction as described. Extracted DNA underwent whole‐exome library construction and somatic mutation analysis as described previously [10]. BAM files were aligned to the hg19 human genome build. Copy number aberrations were quantified and reported for each gene as previously described [11]. Single‐nucleotide variants (SNVs) and small indels were identified using VarScan2 2.3.9 with the minimum variant allele frequency threshold set at 0.01, and the *P* value threshold for calling variants was set at 0.05 to generate variant call format files [12]. All SNVs and indels were annotated with ANNOVAR [13].

### RNA extraction and RT‐PCR

Formalin‐fixed paraffin‐embedded tissues were used to extract tumor RNA. Tumor area was circled by a pathologist in the slide with hematoxylin and eosin staining. RNA was extracted from tissue without hematoxylin and eosin staining with RNeasy FFPE Kit from QIAGEN (Hilden, Germany) according to its protocol. RT‐PCR was performed to amplify Nuclear receptor corepressor (*NCOR1*) exon 19 with primers 5′‐CAGCAGAAGAAACTGAGGAAA‐3′ and 5′‐GGTGGGGGCTCTTCAGTA‐3′. A fragment of glyceraldehyde‐3‐phosphate dehydrogenase (*GAPDH*) as an internal control was amplified with primers P12 (5′‐GACAGTCAGCCGCATCTTCTT‐3′) and P13 (5′‐CAATACGACCAAATCCGTTGAC‐3′). The following PCR conditions were used: 98 °C 2 min; 98 °C 10 s, 55 °C 30 s, 72 °C 1 min, 30 cycles; final extension, 72 °C 5 min.

### Cell culture, RNA interference and western blot

Human PCa lymph node carcinoma of the prostate (LNCaP) cells were grown in RPMI 1640 supplemented with 10% heat‐inactivated FBS, 1% penicillin‐streptomycin in 25‐cm^2^ polystyrene flasks and maintained at 37 °C in a humidified atmosphere with 5% CO_2_. Casodex‐resistant LNCaP‐CR cells were established after prolonged exposure of LNCaP cells to high concentration (100 mm) of Casodex. LNCaP‐CR cells were maintained in RPMI 1640 containing 10% charcoal‐stripped heat‐inactivated FBS, 1% penicillin–streptomycin.

The NCOR1 siRNA and control siRNA (NC) were purchased from GenePharma (Shanghai, China): *NCOR1* siRNA, 5′‐GCAGUAUUGUCCAAAUUAUTT‐3′ and 5′‐AUAAUUUGGACAAUACUGCTT‐3′; NC, 5′‐UUCUCCGAACGUGUCACGUTT‐3′ and 5′‐ACGUGACACGUUCGGAGAATT‐3′. For RNA interference experiments, 5 × 10^4^ LNCaP and LNCaP‐CR cells were seeded with 2 mL of RPMI containing 10% FBS on six‐well plates for 48 h. Then *NCOR1* siRNA (1 μg siRNA/well) was transfected using siRNA‐Mate plus (GenePharma) into cells for 8 h, and then the medium was replaced with fresh medium. Cells were harvested after 48 h, and the RNA was extracted using TRIzol reagent (Thermo Fisher Scientific, Waltham, MA, USA), reverse transcribed to cDNA and amplified by PCR. The *NCOR1* mRNA expression was determined by RT‐PCR. Total protein was prepared by using radio immunoprecipitation assay buffer. Thirty micrograms of whole‐cell extracts was subjected to SDS/PAGE and transferred to a nitrocellulose membrane. After 1‐h block with 5% nonfat dry milk, the membrane was incubated with primary antibodies [NCOR1 (sc‐515934), GAPDH (sc‐365062) from Santa Cruz Biotechnology, Inc., Shanghai, China] for overnight at 4 °C with the concentration of 0.2–0.4 mg·mL^−1^. Then the membrane was incubated for 1 h with the horseradish peroxidase‐conjugated secondary antibody (Amersham Biosciences, Waltham, MA, USA) at 1 : 5000 dilutions. Equal loading and transfer were confirmed by repeat probing for GAPDH. The bands were detected by an enhanced chemiluminescence kit (Amersham Biosciences).

### Reactive oxygen species analysis

A Reactive Oxygen Species (ROS) Assay kit (Beyotime Institute of Biotechnology, ShangHai, China) was used for active ROS detection using the fluorescent probe 2′,7′‐dichlorofluorescin diacetate (DCFH‐DA). The DCFH‐DA reagent must be diluted to 10 μm in serum‐free medium before use. Following the experimental treatments, 10 μm DCFH‐DA was added to cells with subsequent incubation for 30 min at 37 °C in a humidified incubator, and cells were washed three times with serum‐free medium and collected. Then the relative fluorescence intensities were detected.

### Measurement of mitochondrial membrane potential

Measurement of mitochondrial membrane potential (ΔΨm) was evaluated by JC‐1 probe (Beyotime Institute of Biotechnology). LNCaP or LNCaP‐CR cells were cultured in six‐well plates. Then NCOR1 siRNA (1 μg siRNA/well) was transfected. After transfection, the cells were incubated with an equal volume of JC‐1 staining solution (5 μg·mL^−1^) at 37 °C for 20 min and rinsed twice with PBS. Nontreated LNCaP cells were used as negative controls. CCCP‐treated LNCaP cells were used as the positive control. Mitochondrial membrane potentials were monitored by flow cytometer (BD Biosciences, Franklin Lakes, NJ, USA).

### Ethical statement

This study was approved by the Ethical Committee of Jilin Medical University. All subjects gave written informed consent in accordance with the Declaration of Helsinki.

### Statistical analysis

The quantitative data were shown as the mean ± SD. Statistical analysis was performed using the spss 17.0 software (SPSS Inc., USA). The difference between groups was analyzed by one‐way ANOVA. A *P* value < 0.05 was considered to be statistically significant.

## Results

### Landscape of genomic alterations

**Fig. 1:**
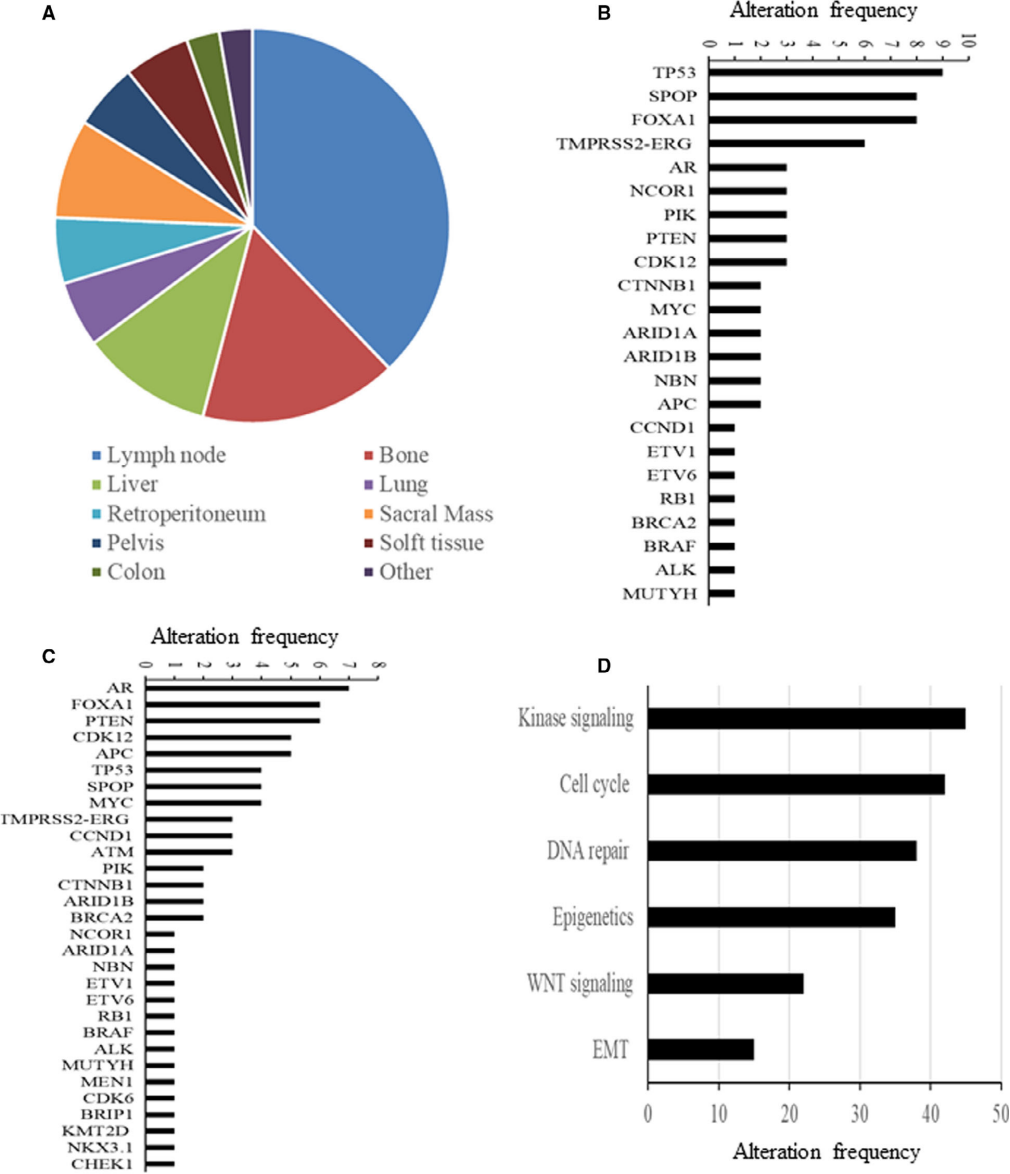
Landscape of genomic alterations. (A) Site distribution of the metastatic cases. (B) Frequency of alteration by gene in primary cases. (C) Frequency of alteration by gene in metastatic cases. (D) Frequency of alteration by pathway. EMT, epithelial mesenchymal transition.

Whole‐exome sequencing was successfully performed on 91 tumors, as well as matched peripheral blood DNA. Of the 91 biopsies, 54 samples are primary tumors derived from patients who were diagnosed with PCa by transurethral resection of the prostate and having a high grade (Gleason score 6 or higher). Of the 37 metastatic biopsies, 38% were lymph node, 15% were bone, 12% were liver and 5% were lung biopsies (Fig. 1A). Median age at diagnosis with PCa was 61 years for primary cases and 62 for metastatic cases. Median age at biopsy of the profiled sample was 64 years for primary cases and 67 for metastatic cases (Table 1).

**Table 1:** Summary of clinical characteristics for 91 patients. PSA, prostate‐specific antigen.

Clinical characteristics			Value	
			Primary cases (*n* = 54)	Metastatic cases (*n* = 37)
Median age at diagnosis (range)			61 (42–85)	62 (40–88)
Median age at biopsy (range)			64 (41–87)	67 (40–89)
Gleason score at diagnosis	I	6 = 3 + 3	14	8
	II	7 = 3 + 4	6	6
	III	7 = 4 + 3	12	6
	IV	8 = 4 + 4	4	2
		8 = 3 + 5	3	2
		8 = 5 + 3	4	5
	V	9 = 4 + 5	4	3
		9 = 5 + 4	5	5
		10 = 5 + 5	0	1
Median PSA at diagnosis (ng·mL^−1^)			13	16

The frequency of genomic alterations, including SNVs, small deletion and insertion (indel), copy number variation and structural variants, was analyzed in both primary and metastatic cases. *TP53*, *SPOP*, *FOXA1*, *AR* and *TMPRSS2–ERG* fusion emerged as the most commonly altered genes in primary cases (Fig. 1B). In metastatic cases, *AR*, *FOXA1*, *PTENC* *CDK12*, *APC* and *TP53* were the most commonly altered genes (Fig. 1C). Consistent with prior reports, a high frequency of genomic alterations in androgen receptor (AR), namely, amplifications and mutations, was confirmed, especially in metastatic cases, with more than 20% of AR amplification (Fig. 1C). More than 30% of cases harbor at least one alteration in kinase signaling, cell‐cycle, epigenetic or DNA repair pathway genes (Fig. 1D).

### *NCOR1* genomic alterations

**Fig. 2:**
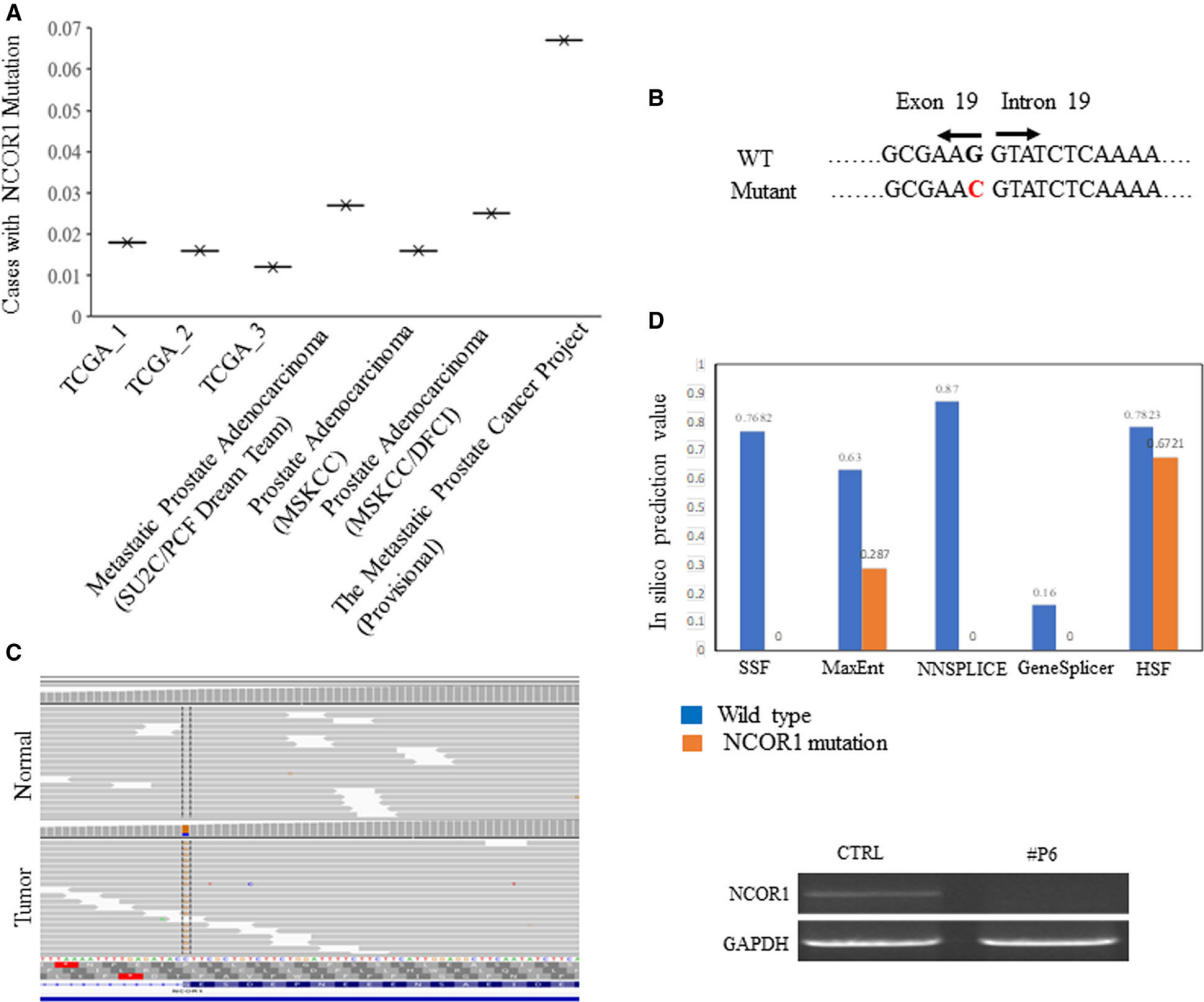
Identification of mutation *NCOR1* c.2182G>C p.Val728Leu. (A) Cases with *NCOR1* mutation from TCGA projects. (B) Boundary of exon 19 and intron 19 of *NCOR1*. (C) Identification of mutation *NCOR1* c.2182G>C p.Val728Leu in the patient by next‐generation sequencing. (D) Mutation *NCOR1* c.2182G>C p.Val728Leu on mRNA splicing by *in silico* prediction tools. SSF, NNSPLICE, NetGene2 and GeneSplicer predict to totally abolish the WT donor site at c.2182. The variant is predicted to significantly damage this WT donor site by MaxEnt (54.5% decreased value). HSF also predicts to weaken this site by the variant (14.1% decreased value). (E) *NCOR1* exon 19 was not detected in the tumor by RT‐PCR.

NCOR1 is a well‐studied corepressor of nuclear receptors, including androgen receptor, involved in repression of their respective target genes. Studies have shown that the PCa response to castration therapies is dependent on functional AR–NCOR1 complexes, and NCOR1 protein levels decline with PCa progression in patients with PCa [14, 15]. In an analysis of recent PCa projects (after 2019) in The Cancer Genome Atlas (TCGA) database, including three TCGA projects, Metastatic Prostate Adenocarcinoma (SU2C/PCF Dream Team), Prostate Adenocarcinoma (Memorial Sloan Kettering Cancer Center), Prostate Adenocarcinoma (Memorial Sloan Kettering Cancer Center/Dana‐Farber Cancer Institute) and The Metastatic PCa Project (Provisional, November 2019), there were a total of 4322 prostate cases. Among them, there were 85 cases with *NCOR1* mutations, with an average of 2.5% (Fig. 2A).

In our cohort, five cases (four primary cases and one metastatic case) were identified with *NCOR1* alterations of four missense mutations and one nonsense mutation (Table 2). The nonsense mutation c.6953C>G p.S2318* was present in a primary case. This case was also identified with the oncogenic *TP53* c.672+1G>T. Although there are no literature and data on the nonsense mutation c.6953C>G p.S2318*, based on its truncated effect on NCOR1, this mutation might be likely oncogenic.

**Table 2:** Summary of the five cases with *NCOR1* genomic alterations. TMB, tumor mutation burden.

Case	TMB	NCoR1	Classification	Other alterations (oncogenic/likely oncogenic)
P6	1.8	c.2182G>C (p.Val728Leu)	Likely oncogenic?	
P16	2.2	c.3056C>T p.P1019L	VUS	
P27	1.6	c.6953C>G p.S2318*	Likely oncogenic	TP53 c.672+1G>T
P33	2.0	c.59A>C p.Y20SC	Likely benign	TP53 c.799C>T p.R267W
M1	2.0	c.1375C>T p.R459C	VUS	AR amplification: 3.9

In case 33, *NCOR1* c.59A>C p.Y20S co‐occurred with likely oncogenic *TP53* c.799C>T p.R267W. Given the fact that of high minor allele frequency (2.24%), *NCOR1* c.59A>C p.Y20S likely would be benign. However, with lack of population data and functional data, both *NCOR1* c.3056C>T p.P1019L and *NCOR1* c.1375C>T p.R459C will be considered as variant with unknown significance (VUS).

The mutation *NCOR1* c.2182G>C (p.Val728Leu) was identified as a somatic mutation in the tumor sample of patient P6 (Fig. 2B,C). In patient P6, the estimated tumor mutation burden for the sample is 1.8 mutations per megabase (mt/Mb). This patient’s cancer does not fall into any subtypes based on molecular features. Neither gene fusions (*ERG*, *ETV1/4* and *FLI1*) nor mutations (*SPOP*, *FOXA1* and *IDH1*) had been identified. There is no driver mutation for PCa in the patient. Loss of heterozygosity was present in the sample. The mutation *NCOR1* c.2182G>C (p.Val728Leu) has not been reported in the literature and in the COMIC database. The sequence changed Val to Leu at position 728. There is a small physicochemical difference between Val and Leu, and *in silico* analysis predicted this to be tolerated on protein structure and function. Further analysis of gene structure revealed that the *NCOR1* c.2182G is the last nucleotide of exon 19 (NM_006311.3). *NCOR1* c.2182G>C probably affects mRNA splicing. *In silico* prediction tools were applied to analyze the potential effect on mRNA splicing by the mutation. splicing site finder (SSF), NNSPLICE, NetGene2 and GeneSplicer predict to totally abolish the WT donor site at c.2182. The variant is predicted to significantly damage this WT donor site by MaxEnt (54.5% decreased value). HSF also predicts to weaken this site by the variant (14.1% decreased value) (Fig. 2D). RT‐PCR confirmed the abnormal mRNA splicing on the *NCOR1* exon 19 in the patient's tumor, suggesting *NCOR1* c.2182G>C (p.Val728Leu) to be likely oncogenic (Fig. 2E).

### Regulation of energy metabolism by NCOR1 in PCa

**Fig. 3:**
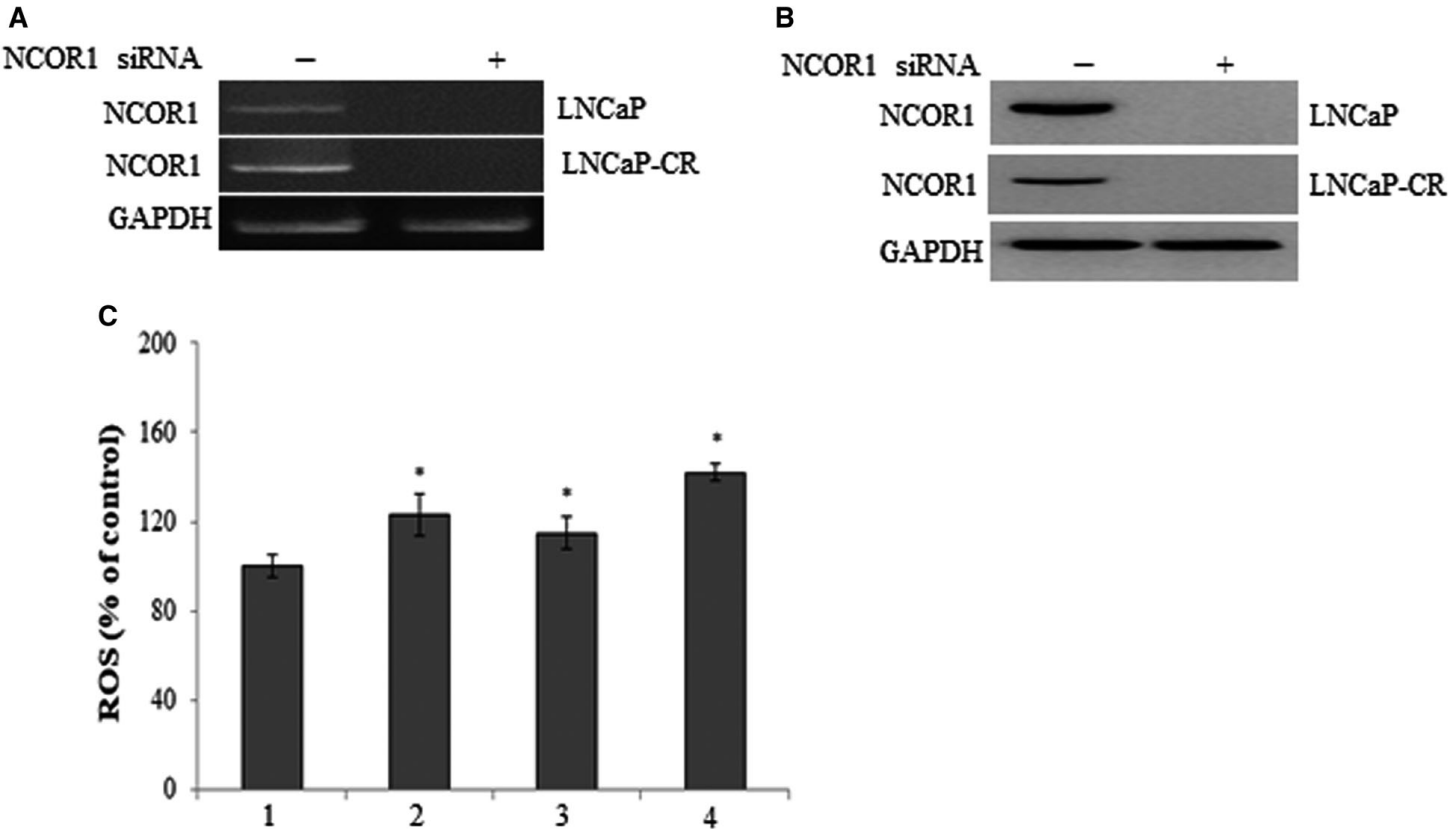
Knockdown of *NCOR1* results in increased ROS level in PCa cells. (A) RT‐PCR showed that NCOR1 was silenced in LNCaP and LNCaP‐CR. (B) NCOR1 was silenced with siRNA in LNCaP and LNCaP‐CR by western blotting. (C) Knockdown of *NCOR1* results in increased ROS level in PCa cells. Column 1, ROS level in LNCaP; column 2, NCOR1 was silenced by siRNA followed by examining ROS level; column 3, ROS level in LNCaP‐CR; column 4, NCOR1 was silenced by siRNA followed by examining ROS level in LNCaP‐CR. Data were indicated as mean ± SD (*n* = 6). *P < 0.05 vs. LNCaP cells. The difference between groups was analyzed by one‐way ANOVA.

**Fig. 4:**
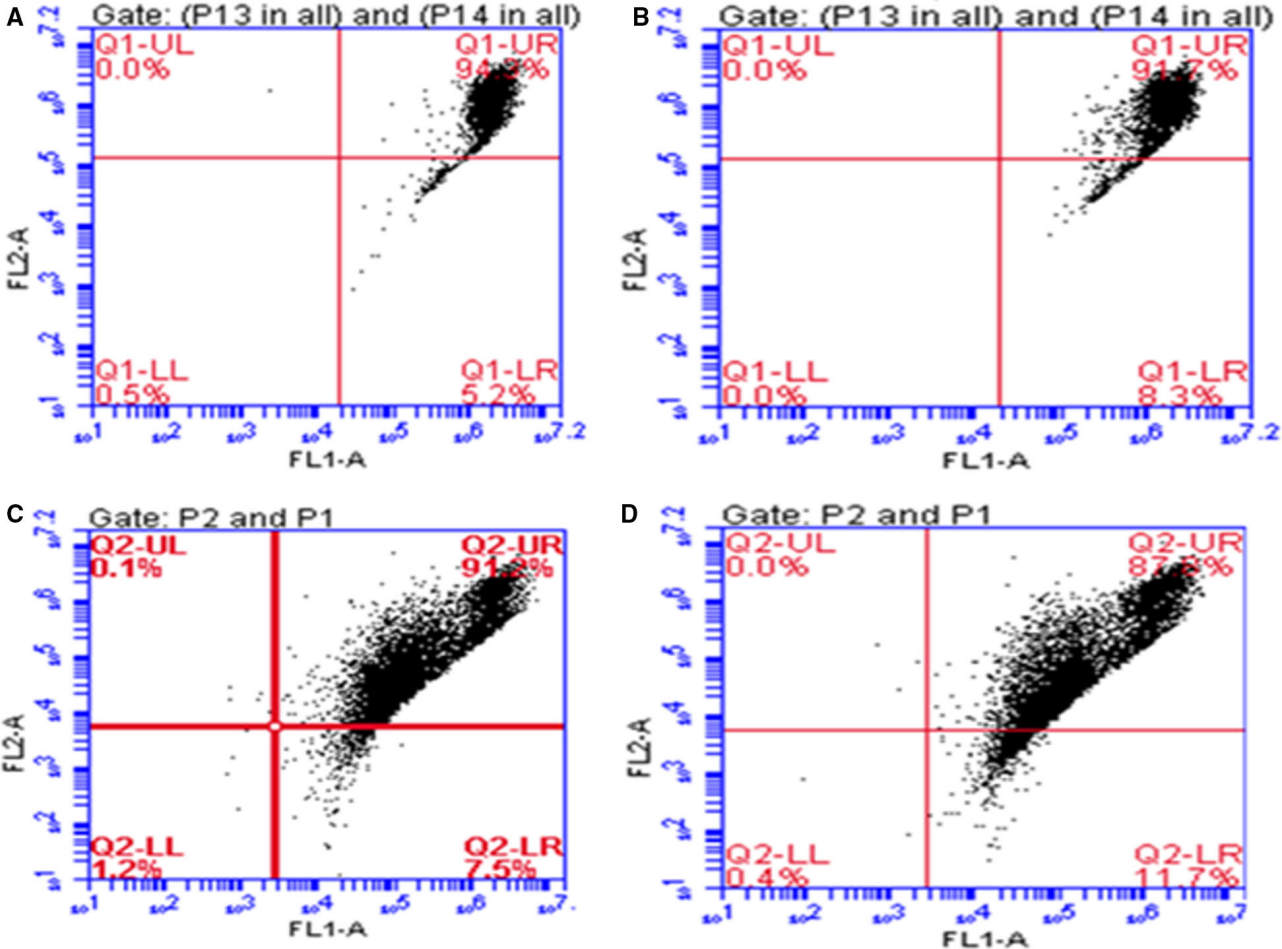
Effect of NCOR1 on ΔΨm in PCa cells. Knockdown of *NCOR1* results in increased ΔΨm of PCa cells. (A) ΔΨm in LNCaP. (B) *NCOR1* was silenced by siRNA followed by examining ΔΨm in LNCaP. (C) ΔΨm in LNCaP‐CR. (D) *NCOR1* was silenced by siRNA followed by examining ΔΨm in LNCaP‐CR.

NCOR1 is a well‐studied corepressor of nuclear receptors, including androgen receptor, involved in repression of their respective target genes. Loss of NCOR1 alters the bicalutamide‐regulated gene expression profile [15]. Besides the role of NCOR1 as corepressor of AR in regulation of PCa progression, it is believed that NCOR1 is also involved in regulation of mitochondrial function, as well as energy metabolism [16]. It is interesting to examine the role of NCOR1 in maintenance of ROS and ΔΨm for PCa progression. ROS is a mediator of intracellular signals and plays an important role in causing apoptotic cell death. *NCOR1* was silenced by siRNA followed by examining ROS in PCa cells LNCaP and LNCaP‐CR (Fig. 3A,B). As shown in Fig. 3C, knockdown of *NCOR1* increases ROS level in both LNCaP and LNCaP‐CR. ΔΨm is an important index on mitochondria function. As shown in Figs 4 and S1, knockdown of *NCOR1* increases ΔΨm in both LNCaP and LNCaP‐CR, and overexpression of *NCOR1* decreases ΔΨm. That indicated collapse of the mitochondrial membrane potential and eventually apoptosis, suggesting the potential role of NCOR1 as suppressor in cancer progression.

## Discussion

Recurrent castration‐resistant PCa (CRPC) is the major cause of mortality of patients with PCa. Understanding the genetic alterations that underlie the development and progression of PCa to CRPC is the major goal of cancer genomics. Molecular characterization and classification of clinically relevant subtypes of PCa are likely to improve prognostic and predictive accuracy for patients. Here we reported comprehensive genomic analysis of 91 patients, including 54 primary and 37 metastatic cases, by whole‐exome sequencing and characterization of NCOR1 as a molecular marker for a possible subtype of PCa.

NCOR1 is a steroid receptor coregulatory protein. This cofactor was shown to repress both agonist‐ and antagonist‐dependent transcriptional activity on ARE‐driven reporters in terms of AR transactivation [17]. Depletion of NCOR1 in PCa cells changed the expression of genes that play significant roles in PCa progression [15]. Five cases (four primary cases and one metastatic case) were identified with *NCOR1* alterations of four missense mutations and one nonsense mutation. In one patient, typical genomic alterations of PCa were not observed except for the mutation *NCOR1*c.2182G>C (p.Val728Leu). *NCOR1* c.2182G is the last nucleotide of exon 19, which is in the WT donor site within the consensus splice‐site regions. Donor and acceptor splice‐site variants typically lead to exon skipping, truncated protein and a loss of protein function. The kinds of variant/mutation are normally pathogenic/likely pathogenic in germline and oncogenic/likely oncogenic in somatics. Whether NCOR1 is an oncogene or tumor suppressor in PCa is still inconclusive. Some studies provided direct evidence *in vivo* that NCOR1 could function as an oncogene via transcription regulation in a mouse model of thyroid cancer [18]. Given the fact of NCOR1 as AR suppressor in PCa progression and an oncogenic role in thyroid cancer, it is reasonable for NCOR1 to act as both oncogene and tumor suppressor.

## Conclusion

Similar to *FOXA1* in the *AR* pathway, genomic alterations in *NCOR1* have been previously reported in primary PCas and CRPC [6, 7]. Besides its role in the AR pathway, NCOR1 plays a key role in mitochondrial gene regulation [19]. Our data showed that NCOR1 is critical in the maintenance of ΔΨm in PCa cells. Knockdown of *NCOR1* increases ΔΨm and eventually results in cell apoptosis. NCOR1 has both nuclear and cytoplasmic fractions that have distinct cellular functions. Nuclear–cytoplasmic shuttling is regulated in part by NCOR1 phosphorylation. How nuclear and cytoplasmic fractions function in mitochondrial metabolism and PCa progression still remains elusive.

## Conflict of interest

The authors declare no conflict of interest.

## Author contributions

WZ and HW conceived and designed the project; LT, LL, LD and YD acquired the data; LZ analyzed and interpreted the data; LZ wrote the paper.

## Supporting information


**Fig. S1.** Overexpression of NCOR1 on ΔΨm of PCa cells. (A) Overexpression of NCOR1 in LNCaP detected by western blotting. (B) NCOR1 was overexpressed followed by examining ΔΨm in LNCaP‐CR.

## Data Availability

Data will be available from the corresponding author upon reasonable request. Please note these are sensitive patient data.
