# Supplementary figures and images for: NCOR1 may be a potential biomarker of a novel molecular subtype of prostate cancer
Source: FEBS Open Bio. 2020 Nov 8;10(12):2678–86. doi: 10.1002/2211-5463.13004 (PMC7714081; doi:10.1002/2211-5463.13004)

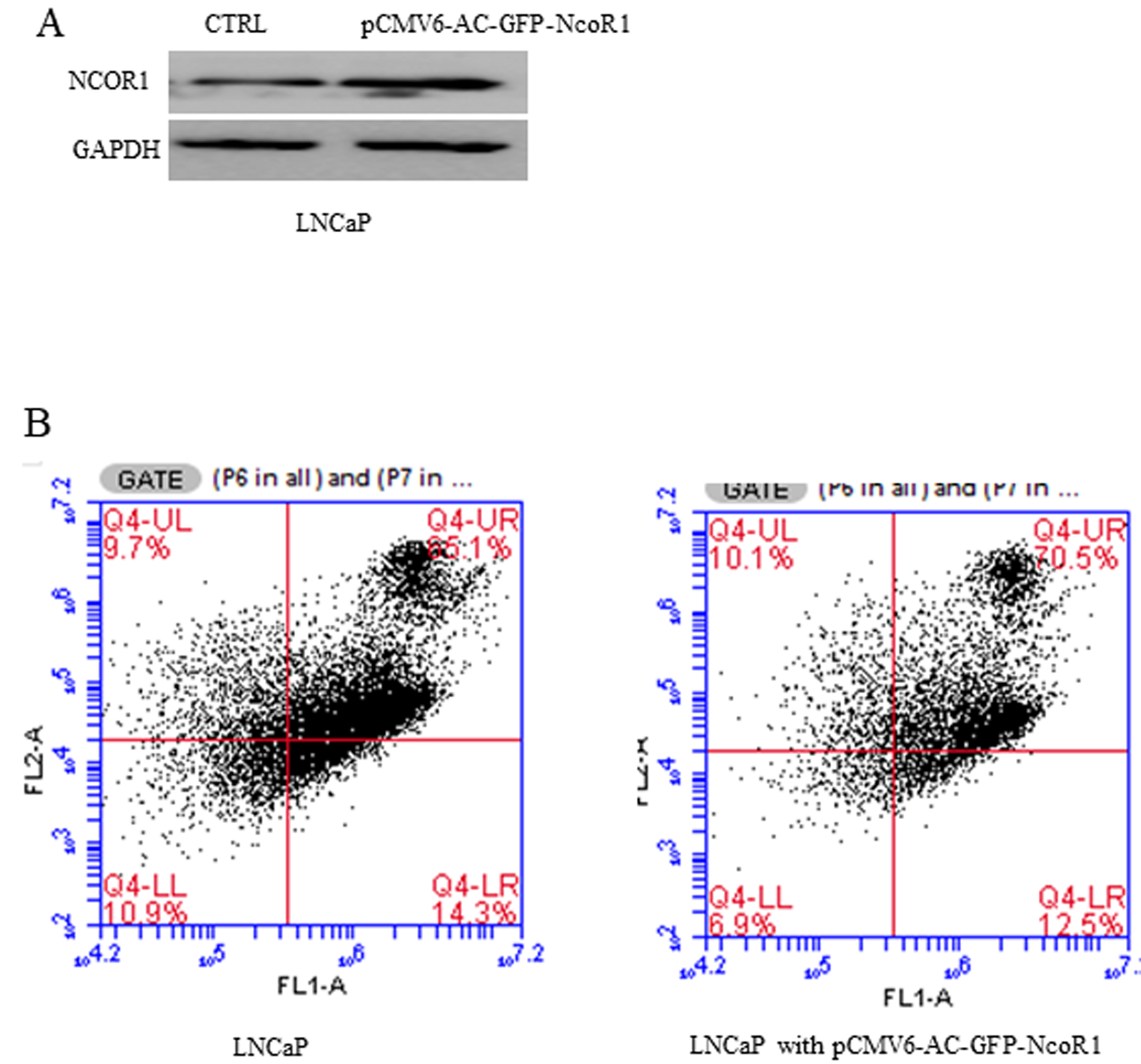

Supplement: Supplementary file 1 — Fig. S1. Overexpression of NCOR1 on ΔΨm of PCa cells. (A) Overexpression of NCOR1 in LNCaP detected by western blotting. (B) NCOR1 was overexpressed followed by examining ΔΨm in LNCaP‐CR. [file FEB4-10-2678-s001.TIF]
